# Multidrug resistance of *Pseudomonas aeruginosa*: do virulence properties impact on resistance patterns?

**DOI:** 10.3389/fmicb.2025.1508941

**Published:** 2025-02-05

**Authors:** Poulomi Saha, Rubaiya Binte Kabir, Chowdhury Rafiqul Ahsan, Mahmuda Yasmin

**Affiliations:** ^1^Department of Microbiology, University of Dhaka, Dhaka, Bangladesh; ^2^Department of Microbiology, Dhaka Medical College, Dhaka, Bangladesh

**Keywords:** multidrug resistance, virulence properties, *Pseudomonas aeruginosa*, nosocomial infection, β-lactamase

## Abstract

**Introduction:**

Patients with nosocomial infections are at risk of multidrug-resistant (MDR) *Pseudomonas aeruginosa* since these bacteria slow down the entire treatment process, increasing the morbidity and mortality of patients staying in hospital. The purpose of the research was to assess the simultaneous presence of multidrug resistance and virulence factors among nosocomial strains of *P. aeruginosa* to evaluate significant association among them.

**Methods:**

One hundred and eight clinical isolates of *P. aeruginosa* were found in a variety of samples taken from patients having nosocomial infection, including wound swabs, pus, sputum, tracheal aspirate, and urine. An antibiogram was performed to investigate the pathogen’s antibiotic sensitivity pattern against 14 widely used antibiotics in Bangladesh. Virulence factors were evaluated, and the presence of ten *β*-lactamase and six virulence genes was analyzed by performing PCR. By using a binary logistic regression test with a 95% confidence interval, the relationship between MDR phenotypes and the virulence attributes was assessed.

**Results:**

The susceptibility rate among the isolates was 70–75% for aminoglycosides (amikacin, gentamicin, netilmicin), 15–20% for cephalosporins (ceftazidime, ceftriaxone), 30–35% for quinolones (ciprofloxacin, levofloxacin), 10–15% for tetracyclines (tigecycline, doxycycline), 15–20% for carbapenem (meropenem), 10–15% for sulfonamide (co-trimoxazole), 5–10% for amoxiclav, and 30–35% for piperacillin/tazobactam. A total of 74.1% of the strains carried metallo-*β*-lactamase (MBL) genes. Among the isolates, 89% showed hemolytic activity, 80–90% produced different pigments such as fluorescein and pyoverdine, 46% were strong biofilm producers, and all the isolates presented different types of motilities (swimming, swarming, and twitching). The virulence genes (*lasB, exoS, toxA, aprA, algD,* and *plcH*) were detected within a range of 60–80% of the isolates.

**Discussion:**

Only the *toxA* gene and twitching motility showed a significant correlation (*p*-value = 0.001 and 0.028, respectively) with multidrug resistance in the clinical *P. aeruginosa* isolates which indicates that it can be used as a drug target to combat these organisms. The high prevalence of MDR strains and their association with virulence factors revealed the potential of the pathogen to cause an infection. The current study advocates for immediate epidemiological surveillance of MDR *P. aeruginosa* strains in Bangladesh to impede the rapid dissemination of this opportunistic pathogen.

## 1 Introduction

*Pseudomonas aeruginosa* is the leading organism responsible for nosocomial infections worldwide. Its capability to produce various potent virulence properties and resistance to various classes of antibiotics has called out the emergence to study the current condition of our country, Bangladesh, regarding the serious issue of multidrug resistance and its dissemination by *P. aeruginosa,* which contributes to in-patient morbidity and mortality (Spagnolo et al., 2021). The ability of multi-drug resistant (MDR) *P. aeruginosa* strains to colonize and grow in settings that other bacterial species cannot, gives them a greater capacity to generate epidemic outbreaks. For an infection to continue and raise patient morbidity in a hospital setting, the bacteria need to be pathogenic and resistant to antibiotics (Martínez and Baquero, 2002). Virulence factors of the pathogen facilitate its persistence and resistance to various environmental stresses including the stringent conditions inside the patients’ body during medication. *P. aeruginosa* possesses genes that enable them to better adapt to their surroundings in this way (Beceiro et al., 2012).

Even though *P. aeruginosa* infections are rarely fatal, the bacteria have developed a high resistance to a number of antimicrobial medicines due to the presence of efflux systems, the production of enzymes that break down antibiotics, reduced outer membrane permeability, and target modifications. As a result, multidrug-resistant strains have become more common (Bassetti et al., 2018; Jácome et al., 2012). By means of intrinsic chromosomally encoded or genetically acquired resistance features, *P. aeruginosa* exhibits the majority of these known resistance mechanisms, hindering the main classes of antibiotics, including aminoglycosides, *β*-lactams, polymyxins, and quinolones (Bassetti et al., 2018). GIM, AIM, SPM, IMP, and VIM are examples of class B metallo-β-lactamases (MBLs), which are also the predominant mechanism of acquired resistance to carbapenems. While varieties of IMP and VIM have been documented globally, GIM, AIM, and SPM have only been observed in a few select geographic areas, such as São Paulo and Germany (Zhao and Hu, 2010). High-risk MDR strains of *P. aeruginosa* are distributed around the world due to a combination of factors including mutation accumulation, horizontally acquired resistance mechanisms, and intrinsic resistance to some antibiotics (del Barrio-Tofiño et al., 2017).

Many virulence factors, including the presence of flagella, pili, and lipopolysaccharides, which are all components of the bacterial structure, along with other factors the bacteria produce and liberate into the adjacent tissue, where the infection expands, such as pigments, proteolytic enzymes, DNases, and toxins, are linked to the pathogenicity of *Pseudomonas* spp. (Pang et al., 2019). Because biofilms are naturally resistant to antimicrobial treatments, biofilm production is another factor contributing to pathogenicity. *P. aeruginosa*’s biofilm exhibits many resistance mechanisms that render it clinically accountable for numerous chronic infections, which are managed by quorum sensing (QS). Moreover, exoenzyme S, exotoxin A, siderophores, quorum sensing system proteins, type III secretory proteins, elastase enzyme, and alginate pigments account for infection initiation and both acute and chronic stages of diseases inside the host.

To lessen the possibility of MDR *P. aeruginosa* strains disseminating, we require information about the isolates’ patterns of antimicrobial susceptibility (Sawa et al., 2014). The World Health Organization (WHO) advocates nosocomial surveillance to enhance infection control practices, particularly in the healthcare sector, and to assist doctors in selecting between directed or empirical treatment (Palavutitotai et al., 2018). To address AMR worldwide, it stressed the significance of creating and sustaining reliable surveillance and reporting systems for AMR genetics, mechanics, and epidemiology (World Health Organization, 2020).

Antimicrobial medicines are commonly provided to patients in Bangladesh; nevertheless, hospitals are currently facing a shortage of pharmaceuticals for patients’ treatment because MDR *P. aeruginosa* is becoming more common. From 2015 to 2018, the number of MDR isolates increased year over year, and by 2019, the increase had nearly doubled from 2015 (Safain et al., 2020). In accordance with the present situation, it is essential to understand the correlation between pathogenicity and antibiotic resistance before making drug choices to eliminate the pathogen. This study intends to convene more knowledge on virulence genes and their relevance to the MDR phenotype of *P. aeruginosa* strains to help design ample therapeutic and control strategies to combat this pathogen.

## 2 Materials and methods

### 2.1 Sample collection and isolation of *Pseudomonas aeruginosa*

The time frame for conducting this study was from February 2023 to December 2023. The inclusion criteria of the patients were being clinically non-responding to antibiotics, and the exclusion criteria were not having nosocomial infections and being outdoor patients or staying at the hospital for less than 7 days. According to patient records, a total of 132 non-duplicate isolates were obtained from distinct samples (wound swab, urine, tracheal aspirate, pus, and sputum) of patients suffering from nosocomial infections (NIs) at Dhaka Medical College Hospital (DMCH), Bangladesh. Information was gathered about the patients’ age, sex, and place of origin for the samples. Every isolate was obtained from a separate patient, and the patients were not associated with epidemic outbreaks. To identify pure colonies of *P. aeruginosa*, the isolates were picked from the MacConkey agar plates, onto which the raw samples were cultured, and then streaked onto Cetrimide agar. *Pseudomonas aeruginosa* isolates were determined by their colony characteristics on Cetrimide agar medium followed by a series of biochemical tests (Gram staining, catalase, oxidase, sugar fermentation, nitrate reduction, motility, indole, urea, citrate utilization, methyl red, and Voges-Proskauer) and molecular identification based on 16S rRNA gene sequencing for further study.

### 2.2 Antimicrobial susceptibility test

The pathogens’ antibiogram was assessed using the disk diffusion method onto Mueller–Hinton agar in accordance with the CLSI (Clinical and Laboratory Standards) guidelines. The antimicrobials tested for resistance included ciprofloxacin (5 μg), co-trimoxazole (25 μg), amikacin (30 μg), ceftazidime (30 μg), piperacillin/tazobactam (100/10 μg), levofloxacin (5 μg), amoxicillin/clavulanic acid (20/10 μg), doxycycline (30 μg), aztreonam (30 μg), ceftriaxone (5 μg), meropenem (10 μg), netilmicin (10 μg), gentamicin (10 μg), and tigecycline (30 μg). The strains were classified as pandrug-resistant (PDR, non-susceptible to all antimicrobial classes), extensively drug-resistant (XDR, non-susceptible to all but two classes), multidrug-resistant (MDR, non-susceptible to ≥3 antimicrobial classes), and low-level drug-resistant (LDR).

### 2.3 Toxin production

Hemolysin and pigment production were evaluated on blood agar (8%) and cetrimide agar plates, respectively. A clear zone around the colonies elucidated the beta-hemolytic activity of the *P. aeruginosa* strains, and the lack of a clear halo was identified as a negative result. Pigments produced on the cetrimide agar were pyoverdine (green), pyocyanin (blue-green), and fluorescein (yellow).

### 2.4 Enzyme production

Proteolytic enzyme (gelatinase) production was studied by stabbing in 4% gelatin media containing test tubes. Hydrolysis (partial or total) of gelatin indicated the production of gelatinase enzyme by the isolates, while a lack of hydrolysis indicated a negative result.

### 2.5 Motility assay

As three types of motility can be detected in *P. aeruginosa* strains, a motility assay was carried out on Luria–Bertani (LB) agar plates using different concentrations of agar to evaluate the following types of motility: twitching (1% agar), swarming (0.5% agar), and swimming (0.3% agar). The media were inoculated with the overnight cultured strains using sterile yellow tips on the surface of the agar to study swarming, by stabbing halfway down the thick agar media for evaluating swimming and for twitching the strains were introduced at the bottom of the agar. All culture plates were kept overnight at a 37°C incubator. To evaluate the twitching motility, the 1% agar plates were flooded with TM developer solution and left for up to 30 min. The colony on the agar surface was scraped away by a sterile loop, and the solution was decanted. The remaining interstitial colony attached to the Petri plate is indicative of the twitching motility of the isolates.

### 2.6 Biofilm production

*P. aeruginosa* strains were inoculated in LB broth on a 96-well microtiter plate to quantify biofilm formation. As a negative control, a sterile broth was used to assure sterility. Biofilm producers were differentiated from non-biofilm producers by measuring their OD values. If the value is ≤ (average OD of negative control +3ơ), the strain was considered non-biofilm producer, and the isolates having OD value > (ANC + 3ơ) were interpreted as biofilm producer strains.

### 2.7 Identification of virulence and MBL genes

DNA extraction of clinical *P. aeruginosa* isolates was carried out by boiling DNA method. The presence of 6 virulence genes (*plcH, lasB, exoS, algD, toxA,* and *aprA*) and 10 MBL genes (*bla*_VIM-2_*, bla*_SPM,_ *bla*_SIM,_ *bla*_DIM,_ *bla*_BIC,_ *bla*_OXA-48,_ *bla*_GIM,_ *bla*_SHV,_ *bla*_DHA_, and *bla*_AIM_) was evaluated by amplifying the genes with specific primers listed in Tables 1, 2, respectively. Previously well-characterized strains were used as controls in this study. PCR conditions were maintained as in the reference articles, respectively. Gel electrophoresis was conducted by running the amplified PCR products for 50 min at 100 V using a 1.5% agarose gel using 1X TBE buffer. The sizes of the amplicons were determined by comparing them with the mobility of the 100 bp and 1 kb + DNA ladder (NEB, UK). The amplicons were visualized, and the gels were photographed using the gel documentation system (Axygen, USA) having a UV trans-illuminator.

**Table 1:** Oligonucleotide primers used in amplifying of virulence genes.

Target gene	Primers	Primer sequence (5` → 3`)	Amplicon size (bp)	References
*algD*	AlgD-F	ATG CGA ATC AGC ATC TTT GGT	1,310	Lanotte et al. (2004)
	AlgD-R	CTA CCA GCA GAT GCC CTC GGC		
*aprA*	AprA-F	GTC GAC CAG GCG GCG GAG CAG ATA	993	Schmidtchen et al. (2001)
	AprA-R	GCC GAG GCC GCC GTA GAG GAT GTC		
*lasB*	LasB-F	GGA ATG AAC GAG GCG TTC TC	300	Lanotte et al. (2004)
	LasB-R	GGT CCA GTA GTA GCG GTT GG		
*plcH*	PlcH-F	GAA GCC ATG GGC TAC TTC AA	307	Lanotte et al. (2004)
	PlcH-R	AGA GTG ACG AGG AGC GGT AG		
*exoS*	ExoS-F	CTT GAA GGG ACT CGA CAA GG	504	Lanotte et al. (2004)
	ExoS-R	TTC AGG TCC GCG TAG TGA AT		
*toxA*	ToxA-F	GGA GCG CAA CTA TCC CAC T	150	Michalska and Wolf (2015)
	ToxA-R	TGG TAG CCG ACG AAC ACA TA		

**Table 2:** Oligonucleotide primers used in amplifying MBL genes.

Target Gene	Primers	Primer sequence (5` → 3`)	Amplicon size (bp)	References
*bla*_VIM-2_	VIM-2-F	ATTGGTCTATTTGACCGCGTC	801	
	VIM-2-R	TGCTACTCAACGACTGAGCG		
*bla*_SPM_	SPM-F	AAAATCTGGGTACGCAAACG	271	Poirel et al. (2011)
	SPM-R	ACATTATCCGCTGGAACAGG		
*bla*_AIM_	AIM-F	CTGAAGGTGTACGGAAACAC	322	
	AIM-R	GTTCGGCCACCTCGAATTG		
*bla*_SIM_	SIM-F	TACAAGGGATTCGGCATCG	570	
	SIM-R	TAATGGCCTGTTCCCATGTG		
*bla*_BIC_	BIC-F	TATGCAGCTCCTTTAAGGGC	537	
	BIC-R	TCATTGCGGTGCCGTACAC		
*bla*_GIM_	GIM-F	TCGACACACCTTGGTCTGAA	477	
	GIM-R	AACTTCCAACTTTGCCATGC		
*bla*_SHV_	SHV-F	TTTATGGCGTTACCTTTGACC	1,050	Mathlouthi et al. (2016)
	SHV-R	ATTTGTCGCTCTTTACTCGC		
*bla*_DHA_	DHA-F	AACTTTCACAGGTGTGCTGGGT	405	Liu and Liu (2016)
	DHA-R	CCGTACGCATACTGGCTTTGC		
*bla*_OXA-48_	OXA-48-F	GCGTGGTTAAGGATGAACAC	438	Poirel et al. (2011)
	OXA-48-R	CATCAAGTTCAACCCAACCG		
*bla*_DIM_	DIM-F	GCTTGTCTTCGCTTGCTAACG	699	
	DIM-R	CGTTCGGCTGGATTGATTTG		

### 2.8 Statistical analysis of data

Using binary logistic regression analysis, with a 95% confidence interval at a 5% significance level, the relationship between MDR phenotypes and the virulence factors as well as epidemiological and clinical factors was assessed. The statistical program SPSS version 25 (IBM, New York, USA) was used for the analysis.

## 3 Results

### 3.1 Identification of clinical isolates of *Pseudomonas aeruginosa*

Initially collected 132 isolates taken from patients aged 25–68 years were undergone biochemical tests (Supplementary Table S1), and of them, 108 isolates were plausibly identified as *Pseudomonas aeruginosa.* Twenty-five selected isolates out of 108 were further identified as *P. aeruginosa* using molecular techniques based on 16S rRNA gene sequencing (Supplementary Table S2). Among the patients, 46% were male and 54% were female having low fever and UTI.

### 3.2 Antibiotic resistance profile of *Pseudomonas aeruginosa* isolates

**Figure 1:**
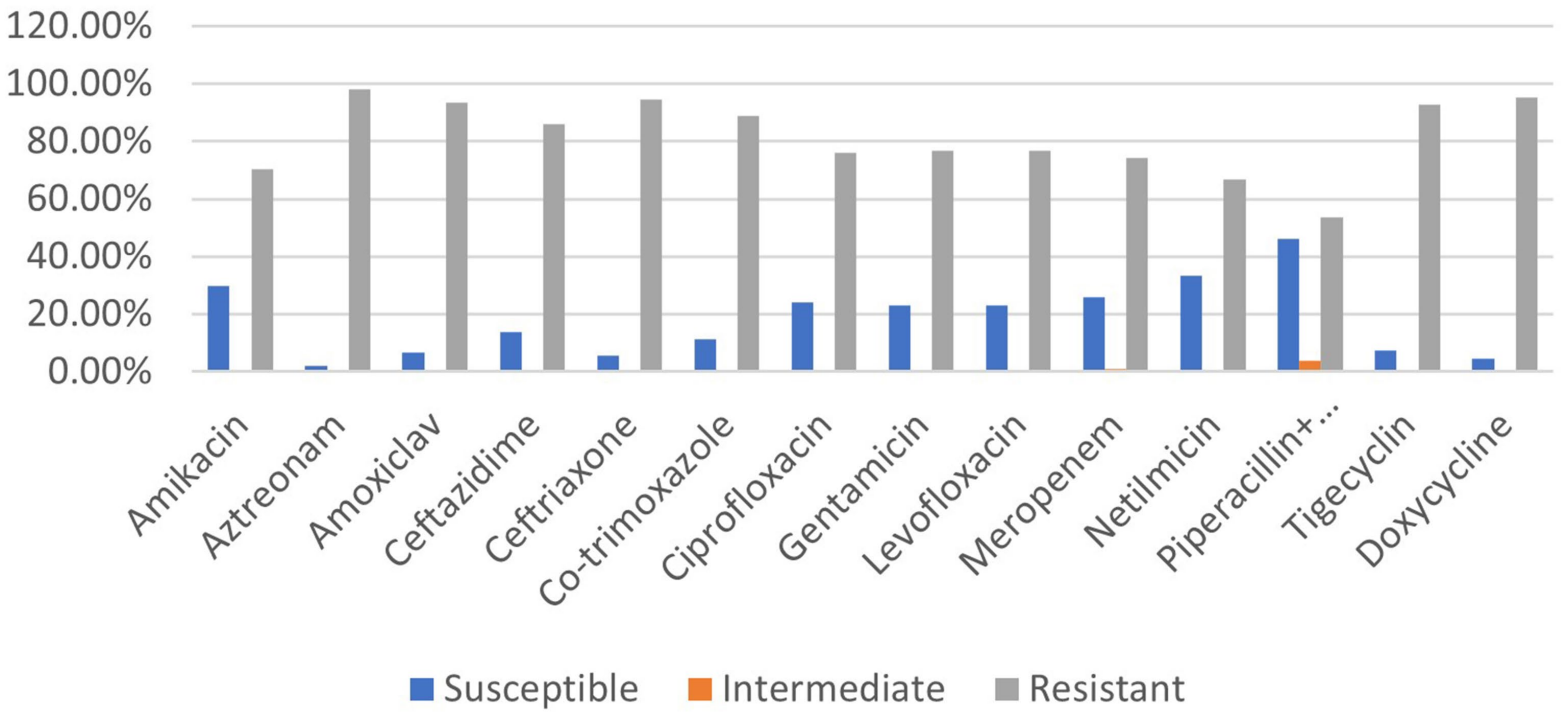
Prevalence rate of clinical *P. aeruginosa* strains resistant to different classes of antibiotics.

The prevalence rate of antibiotic resistance was found to be extremely high (53.7–98.1%) for all antimicrobial agents tested among the 108 clinical isolates of *P. aeruginosa*. In comparison with the other 13 antibiotics (doxycycline, meropenem, ceftazidime, tigecycline, levofloxacin, aztreonam, amoxiclav, netilmicin, gentamycin, co-trimoxazole, ceftriaxone, ciprofloxacin, and amikacin), *P. aeruginosa* strains exhibited significantly less resistance (53.7%) against piperacillin–tazobactam. Comparing the isolates to the other antibiotics revealed the highest level of resistance to aztreonam (98.1%) which belongs to the class monobactam (Figure 1). Amoxiclav (93.5%), ceftriaxone (94.4%), and doxycycline (95.4%) are the remaining drugs that came next in order of resistance (Figure 1).

Out of 108 isolates, only 1 was found to be resistant to each of the 14 antibiotics across 8 distinct classes. The other 107 strains were found to be resistant to at least one of the antibiotics tested. Of them, one strain was resistant to two antibiotics of different classes, while two isolates were found to be resistant to only one antibiotic. These strains of *P. aeruginosa* (2.7%) fall into the low-level drug-resistant (LDR) category. A total of 27.8% of the isolates were classified as MDR strains because they exhibited resistance to at least three classes of antibiotics. Since 58.3% showed no resistance to at least two classes of antibiotics, they were classified as extensively drug-resistant (XDR). A total of 10.2% of the clinical *P. aeruginosa* strains tested positive for every antibiotic and thus fell under the category of pandrug-resistant (PDR).

### 3.3 Prevalence of β-lactamase genes in *Pseudomonas aeruginosa* isolates

**Figure 2:**
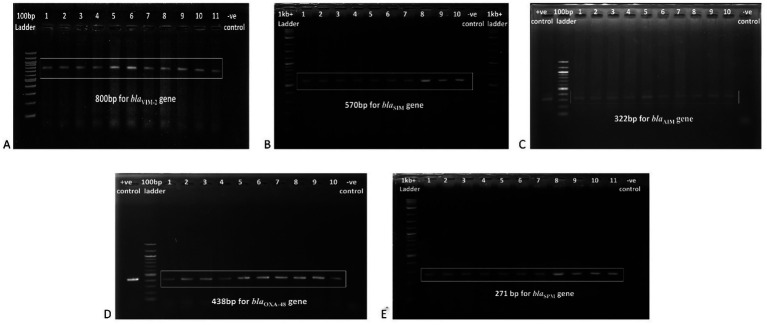
Gel electrophoresis (1.5% agarose) of the beta-lactamase genes present in the *P. aeruginosa* isolates, **(A)** *bla*_VIM-2_ gene at 800 bp, **(B)** *bla*_SIM_ gene at 570 bp, **(C)** *bla*_AIM_ gene at 322 bp, **(D)** *bla*_OXA-48_ gene at 438 bp, and **(E)** *bla*_SPM_ gene at 271 bp.

**Figure 3:**
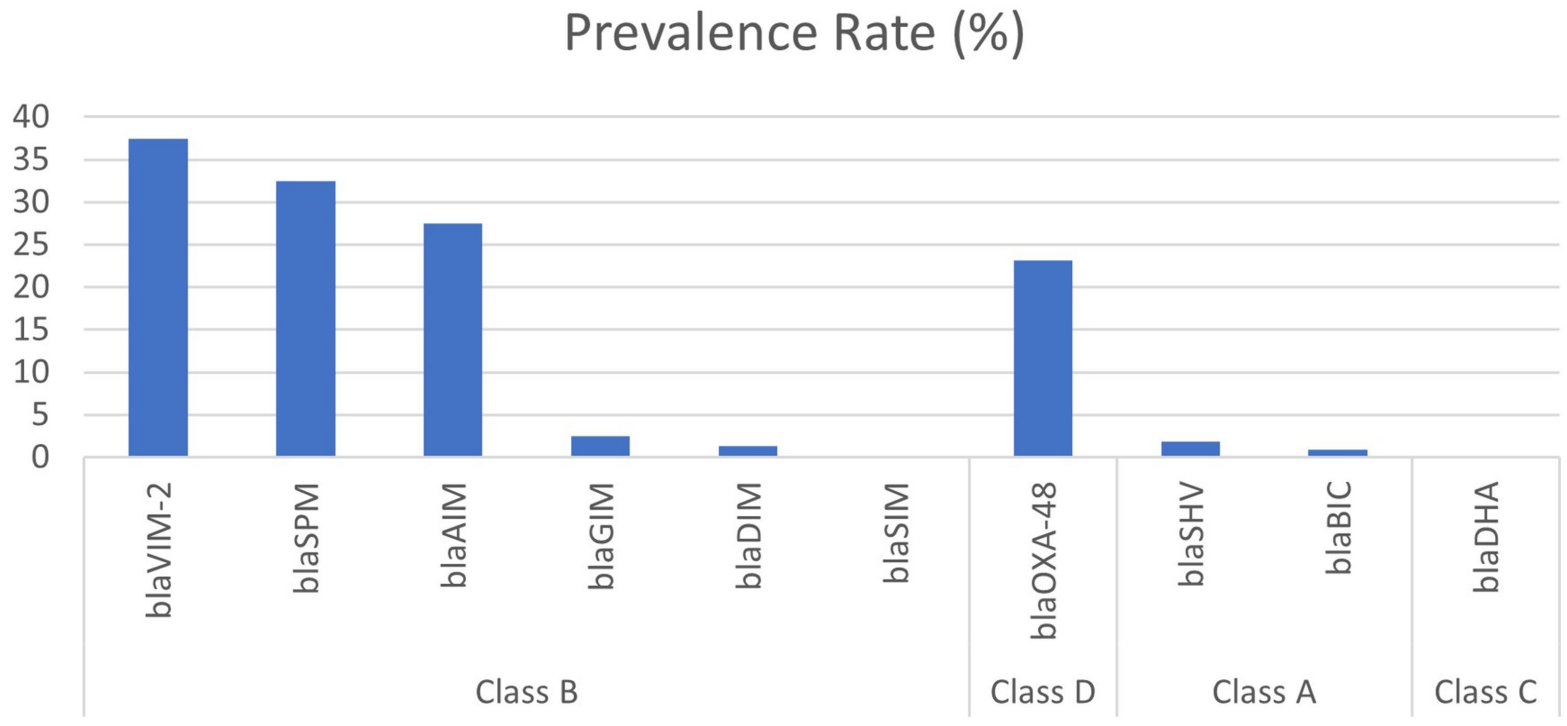
Prevalence of different classes of beta-lactamase gene-producing clinical isolates of *Pseudomonas aeruginosa.*

PCR and gel electrophoresis were performed to detect the presence of beta-lactamase genes in the *P. aeruginosa* isolates (Figure 2). The rates of different classes of beta-lactamase (Ambler classes A, B, C, and D) gene production among the clinical *Pseudomonas aeruginosa* isolates are shown in Figure 3. Among the 10 beta-lactamase genes, 6 are under class B (*bla*_VIM-2_*, bla*_SPM,_ *bla*_SIM,_ *bla*_DIM,_ *bla*_GIM,_ and *bla*_AIM_), 2 of them are classified as class A (*bla*_BIC_ and *bla*_SHV_), 1 is from class D (*bla*_OXA-48_), and another 1 is from class C (*bla*_DHA_). Most of the isolates (74.1%) were detected positive for MBL genes, which are under class B beta-lactamases. Among them, 37.5% harbored the *bla*_VIM-2_ gene, followed by *bla*_SPM_ (32.5%), *bla*_AIM_ (27.5%), and *bla*_GIM_ (2.5%) genes. Only one isolate was found to be positive for the *bla*_DIM_ gene, while the *bla*_SIM_ gene was not identified in any of the isolates. The highest rate of MBL gene production was found in strains isolated from wound swabs (50.1%).

Class D beta-lactamase (*bla*_OXA-48_) gene was found in 23.2% of the *P. aeruginosa* isolates, followed by class A beta-lactamase genes (*n* = 3, 2.7%). No isolates were found to harbor the class C beta-lactamase gene.

### 3.4 Identification of virulence factors in *Pseudomonas aeruginosa* isolates

**Figure 4:**
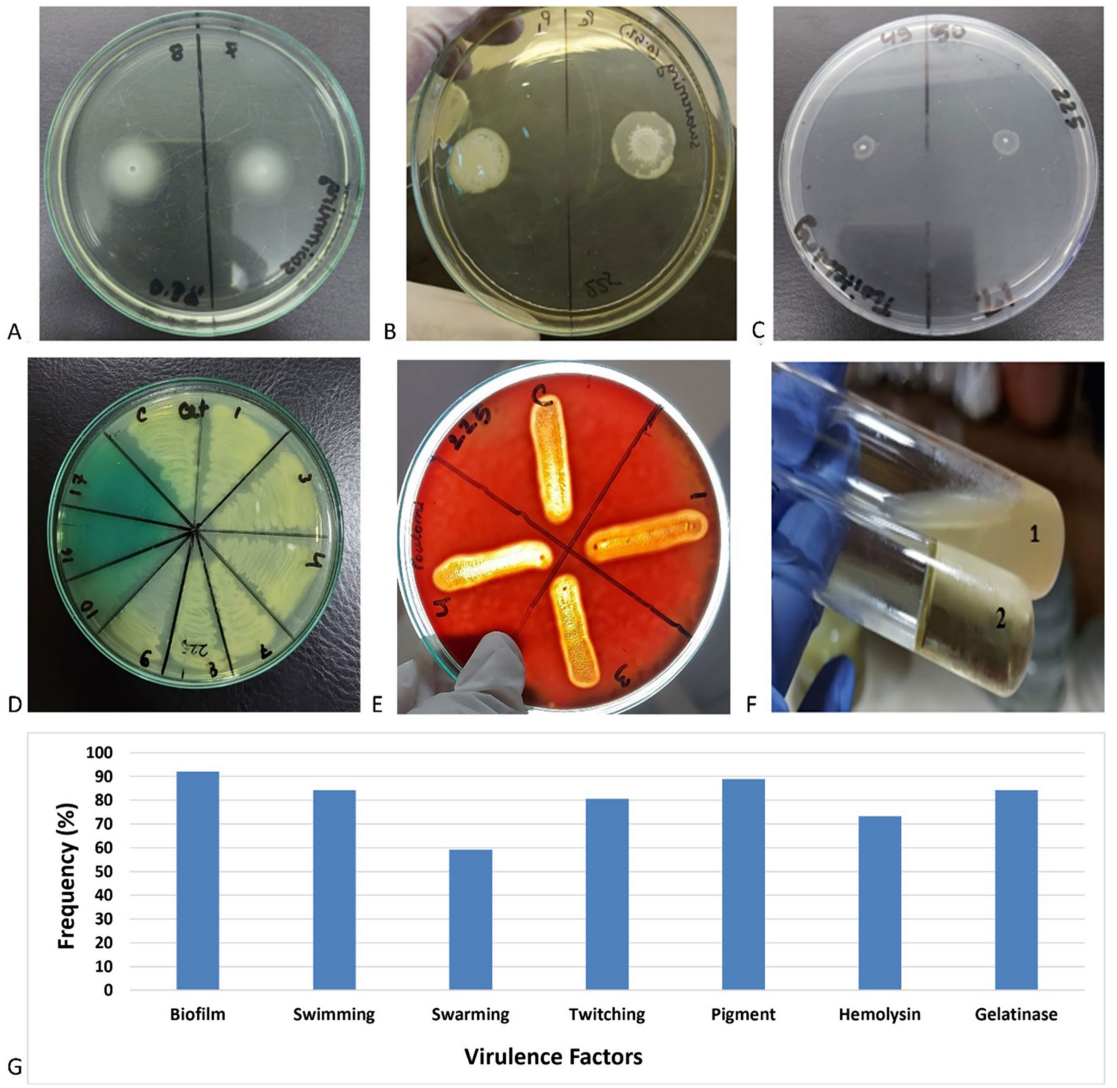
Examples of the experiments for the production of **(A)** swimming, **(B)** swarming, **(C)** twitching, **(D)** pigments, **(E)** hemolysins, **(F)** gelatinase (1: gelatinase positive and 2: gelatinase negative), and **(G)** frequency of different virulence factors in clinical *Pseudomonas aeruginosa* isolates.

All but two isolates of *P. aeruginosa* showed different types of motilities, and most of the strains (84.26%) were capable of swimming which requires rotating flagella, followed by twitching (80.56%) which indicates the existence of type IV pili and swarming (59.25%). The second most frequently found virulence factor synthesized by the *P. aeruginosa* isolates was pigment (88.89%). Among the pigment-producing strains, 70.83% produced fluorescein and subsequently pyoverdine (28.12%) and pyocyanin (9.37%). A total of 7.30% of the strains produced both pigments, fluorescein, and pyoverdine. Gelatinase production was marked in 84.26% of the *P. aeruginosa* strains followed by hemolysin (73.15%) and biofilm formation (67.59%) (Figure 4).

### 3.5 Identification of virulence genes

**Figure 5:**
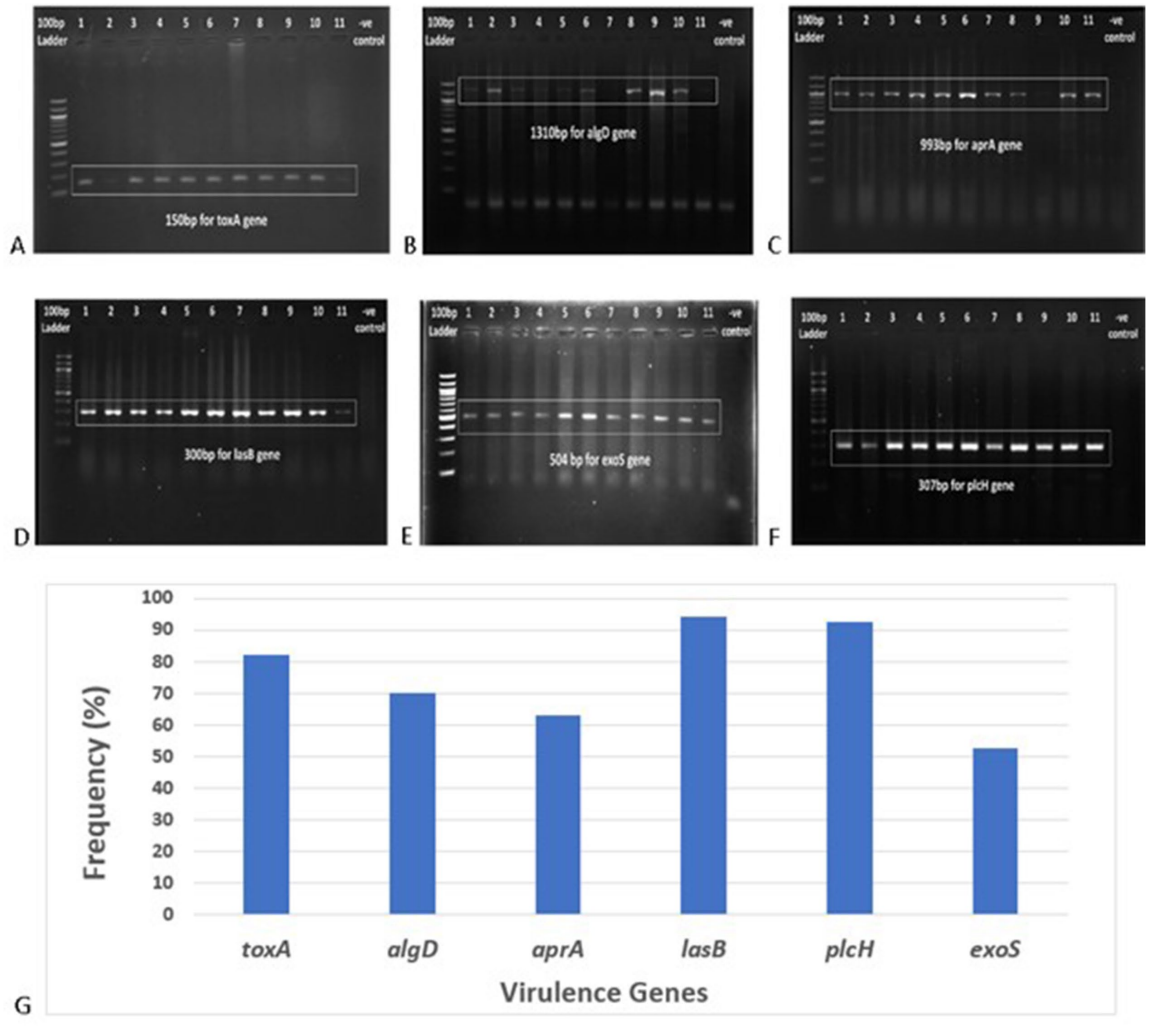
Gel electrophoresis (1.5% agarose) of the virulence genes present in *P. aeruginosa* isolates, **(A)** *toxA* gene at 150 bp, **(B)** *algD* gene at 1310 bp, **(C)** *aprA* gene at 993 bp, **(D)** *lasB* gene at 300 bp, **(E)** *exoS* gene at 504 bp, **(F)** *plcH* gene at 307 bp, and **(G)** frequency of different virulence genes in clinical *Pseudomonas aeruginosa* isolates.

Among the six virulence genes, *plcH* and *lasB* were most frequent among the *P. aeruginosa* isolates (92.59 and 94.44%, respectively) (Figure 5). A total of 82.41% of the strains harbored the *toxA* gene, and the prevalence of the *toxA* gene among MDR *P. aeruginosa* strains was 90.27%. A total of 70.37% of the isolates were found to be positive for the *algD* gene. A total of 62.96 and 55.56% of the isolates were found positive for *aprA* and *exoS* genes, respectively. In addition, 18.52% of the strains were positive for all the virulence genes tested. The frequency of the virulence genes among different sample types is depicted in Table 3.

**Table 3:** Prevalence of different virulence genes among clinical samples.

Clinical sample	*toxA*	*algD*	*aprA*	*lasB*	*plcH*	*exoS*
Pus	33%	67%	67%	67%	100%	100%
Sputum	100%	67%	33%	100%	100%	100%
Tracheal Aspirate	86%	86%	29%	100%	86%	100%
Wound Swab	100%	71%	76%	100%	100%	86%
Urine	100%	89%	56%	100%	100%	89%

### 3.6 Correlation of factors with MDR phenotypes

**Figure 6:**
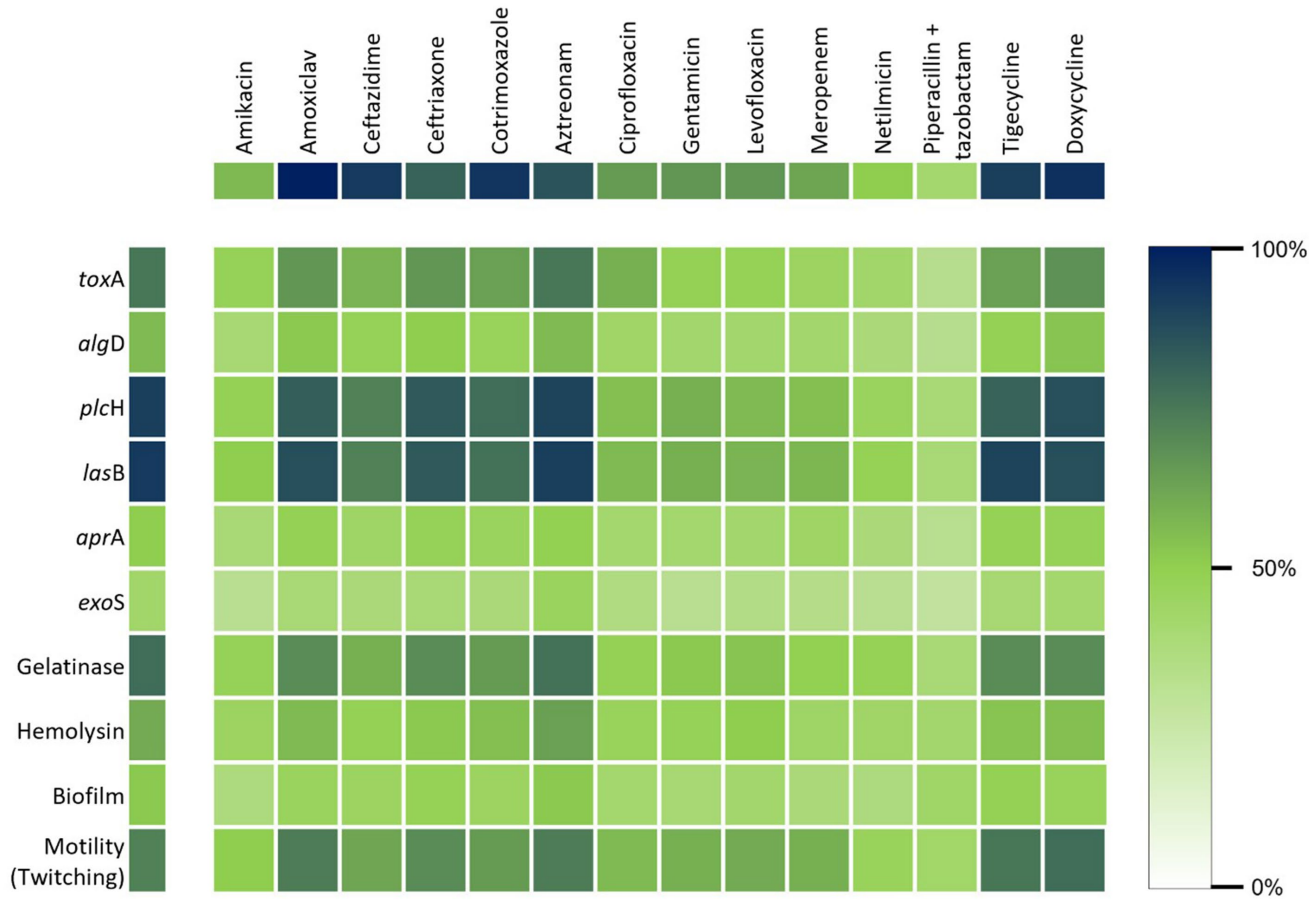
Heat map shows the correlation between the virulence properties and the resistance of *P. aeruginosa* against the commonly used antibiotics.

Biofilm formation, gelatinase activity, hemolysin, and pigment production, which are known virulence factors for *P. aeruginosa,* were found to have no statistically significant correlation with the MDR pattern of *P. aeruginosa* isolates. Among the three types of motilities, swimming and swarming had no association while twitching appeared to be significantly associated (*p* < 0.05) with the level of antibiotic resistance. MDR strains were statistically more likely to harbor the *toxA* gene than the other five when analyzing the virulence genes (Figure 6).

In the statistical model developed for determining the virulence factors as the risk factors for the MDR pattern, the strongest predictor of multidrug resistance was the *toxA* gene, recording an odds ratio (OR) of 8.45 (Table 4). This indicated that the *P. aeruginosa* isolates containing the *toxA* gene were 8.45 times more likely to be multidrug-resistant than the isolates lacking the *toxA* gene. In addition, the odds ratio for twitching motility was 3.31, which indicated that the existence of this type of motility, which is acquired by type IV pili, enhanced the risk of the strains to be MDR by 3.31 times, as found in the statistical analysis. Epidemiological clinical factors such as patient’s age, sex, and types of samples were analyzed and not found to be statistically related to antibiotic resistance.

**Table 4:** Statistical association among different virulence factors and MDR/XDR strains of *Pseudomonas aeruginosa.*

Virulence factors	XDR/MDR			Total Isolates	p-value	OR	95% CI
		−	+				
Biofilm	−	12	35	47	0.262	1.737	0.6–4.5
	+	21	40	61			
Swimming	−	8	8	16	0.183	2.257	0.6–7.4
	+	29	63	92			
Swarming	−	11	32	43	0.255	0.597	0.2–1.4
	+	24	41	65			
Twitching	−	11	10	21	0.028	3.305	1.1–9.5
	+	25	62	87			
Pigment	−	5	8	13	0.865	1.130	0.2–4.6
	+	30	65	95			
Hemolysin	−	11	18	29	0.654	1.246	0.4–3.2
	+	25	54	79			
Gelatinase	−	8	8	16	0.565	1.468	0.3–5.4
	+	29	63	92			
*exoS*	−	2	3	5	0.339	0.402	0.2–1.5
	+	37	66	103			
*toxA*	−	17	10	27	0.001	8.45	2.8–24.8
	+	16	65	81			
*plcH*	−	2	6	8	0.238	0.302	0.1–2.2
	+	35	65	100			
*lasB*	−	4	2	6	0.400	2.131	0.3–12.4
	+	33	69	102			
*aprA*	−	10	28	38	0.352	1.857	0.5–6.8
	+	15	55	70			
*algD*	−	13	29	42	0.619	1.297	0.4–3.6
	+	22	44	66			

CI: confidence interval; OR: odds ratio.

## 4 Discussion

The global dissemination of antibiotic resistance among Gram-negative bacteria is a burning concern for the medical science as the whole world is heading to the pre-antibiotic era. *Pseudomonas aeruginosa* plays a major role in this concern as this opportunistic pathogen causes nosocomial infection in in-patients. The present study demonstrates an alarming state of antibiotic resistance where the *P. aeruginosa* strains have become resistant to almost all antipseudomonal antibiotics. Even carbapenems, which are considered last-line antibiotic drugs, are not able to cure infections sustained by *P. aeruginosa*. In this study, 74.08% of the strains were resistant to carbapenem, and 86.11 to 93.44% of the strains were resistant to third-generation cephalosporins whereas susceptibility of *Pseudomonas* spp. to third-generation cephalosporins ranged from 43.3 to 46.7% in the last decade in Bangladesh (Begum et al., 2013). However, the antibiogram revealed combination drugs as the most effective drug (46.29%) among all antibiotics used against *P. aeruginosa* strains followed by netilmicin (33.33%).

A total of 66.67% of the strains showed resistance to all but two groups of antibiotics used in this study, which are regularly prescribed in Bangladesh for the treatment of nosocomial infections caused by *P. aeruginosa*. These clinical isolates of *P. aeruginosa* are now classified as extensively drug-resistant (XDR) strains rather than multidrug-resistant (MDR). If unchecked use of antibiotics goes on, in the near future all *P. aeruginosa* strains will become pandrug-resistant and patients will die of secondary nosocomial infections rather than of the disease they went to hospital for treatment.

The prevalence of beta-lactamase genes of the four Ambler classes (classes A–D) was evaluated in this study, which showed the highest rampancy for MBL genes (class B). The *bla*_VIM-2_ (37.5%), *bla*_SPM_ (32.5%), and *bla*_AIM_ (27.5%) are the predominant MBLs among the clinical isolates of *P. aeruginosa,* followed by class D (23.2%) and class A (2.8%) beta-lactamase genes. In a study in 2020, the prevalence rate of the *bla*_VIM-2_ gene in clinical *P. aeruginosa* isolates in China was 18.8%, followed by *bla*_SIM_ (6.0%) and *bla*_SPM_ (4.0%) (Wang and Wang, 2020), while in this study 32.5% strain was found to harbor the *bla*_SPM_ gene but no strain was positive for *the bla*_SIM_ gene. The frequency of the *bla*_GIM_ gene was found to be 2.5%, which is greater than that in China.

In another study in South Africa, the *bla*_SHV_ gene was found in 69.5% of *P. aeruginosa* strains (Hosu et al., 2021), in contrast to 2.8% of class A beta-lactam producers in our study. In this study, all strains were negative for class C beta-lactamase genes but in a study of Iran 33.0% of *P. aeruginosa* isolates were found to be positive for class C beta-lactamase genes (Sharifi et al., 2019). Our study depicts that in Bangladesh, class B or MBL genes have the highest prevalence followed by class D and class A beta-lactamases.

Virulence factors such as pigment, hemolysin, biofilm, and motility assist the pathogen in developing an infection inside the host body. In the present study, the most frequently found virulence factor was motility, and twitching motility was directly related to MDR. This indicates the presence of type IV pili is a prominent factor for the pathogen to be virulent and antibiotic-resistant at the same time. Type IV pili is used by *P. aeruginosa* not only for surface motility but also as a sensor to modulate virulence and surface-induced gene expression (Persat et al., 2015).

Gelatinase secreted by the pathogen can degrade a broad range of host substrates such as collagen and complement and delay the airway epithelial wound repair by altering the actin cytoskeleton (de Bentzmann et al., 2000). A total of 84.26% of the strains were found to produce gelatinase enzyme.

Pigments such as pyoverdine, pyocyanin, and fluorescein were produced by 88.89% of the strains. Pyocyanin is secreted by T2SS and can decline lung function by its free radical and pro-inflammatory effects (Hall et al., 2016). Pyoverdine is a fluorescent pigment that is a potent iron (III) scavenger and acts as a siderophore satisfying the pathogen’s absolute iron requirement (Dauner and Skerra, 2020). Beta-hemolysin production plays a major role in disseminating infection by creating pores in cell membranes and assisting the pathogen to extend the wounds.

Biofilm production provides the pathogen with high resistance to host defense, antibiotics, and disinfectants leading to the establishment of chronic infections in in-patients (Lee and Yoon, 2017). Biofilm formation was seen in 67.59% of the isolates, and 41.1% of them were strong biofilm producers followed by 26.03% moderate and 32.88% weak biofilm-forming strains.

In this study, six virulence genes were evaluated to observe their prevalence in the nosocomial strains of *P. aeruginosa*. Then, association of these genes with the antibiotic resistance profile of the test isolates was measured statistically.

All the virulence factors and virulence genes were under the null hypothesis that there is no significant correlation between the MDR traits and virulence factors of the clinical *P. aeruginosa* isolates, except the *toxA* gene and twitching motility. This gene was found to be associated with the MDR traits of nosocomial strains of *P. aeruginosa*, assessed statistically with binary regression analysis. As shown in Table 4, biofilm had no significant correlation with the MDR pattern, although it is well-known for the pathogenesis of clinical *P. aeruginosa*.

Multidrug resistance of *P. aeruginosa* is an alarming concern in Bangladesh. Our knowledge to date about their virulence capability is scanty. Our findings reveal that multidrug resistance and virulence factors are mutually complementary while infecting and causing disease in the patient’s body. Although we have analyzed various virulence properties among MDR strains, most of them were not associated with the resistance pattern. We found that the *toxA* gene and twitching motility are significantly associated with the MDR pattern. Therefore, they can be utilized as new therapeutic targets to treat nosocomial infections caused by MDR *P. aeruginosa* (Alabdali, 2021).

## Data Availability

The original contributions presented in the study are included in the article/Supplementary material, further inquiries can be directed to the corresponding author.
