# Supplementary material for: Multidrug resistance of Pseudomonas aeruginosa: do virulence properties impact on resistance patterns?
Source: Front Microbiol. 2025 Feb 5;16:1508941. doi: 10.3389/fmicb.2025.1508941 (PMC11865748; doi:10.3389/fmicb.2025.1508941)
Supplement: Supplementary file 1 [file Data_Sheet_1.PDF]

Supplementary Table 1: Biochemical Identification of Bacterial Isolates

SL no	Isolate ID	Patient's ID	Sample type	Colony Characteristics in Cetrimide agar		Gram Staining	Catalase	Oxidase	KIA			Citrate	MIU		NR	MR	VP	Indole	Genus Identification
				Pigment	UV				S	B	G		U	M					
1	MPY_0001	DMC2301001	Tracheal Aspirate	Yellow, mucoid	Fluorescence	-	+	+	R	R	-	+	-	+	+	-	-	-	*Pseudomonas spp* (possibly *P. aeruginosa*)
2	MPY_0002	DMC2301003	Urine	Yellow, mucoid	Fluorescence	-	+	+	R	R	-	+	-	+	+	-	-	-	*Pseudomonas spp* (possibly *P. aeruginosa*)
3	MPY_0003	DMC2301004	Wound Swab	Yellow, mucoid	Fluorescence	-	+	+	R	R	-	+	-	+	+	-	-	-	*Pseudomonas spp* (possibly *P. aeruginosa*)
4	MPY_0004	DMC2301005	Urine	No Pigment	No Fluorescence	-	+	-	R	Y	-	-	+	-	+	+	-	+	*Proteus spp*
5	MPY_0005	DMC2301006	Sputum	No Pigment	No Fluorescence	-	+	-	Y	Y	+	+	-	-	+	-	+	-	*Enterobacter app*
6	MPY_0006	DMC2301007	Tracheal Aspirate	Yellow, mucoid	Fluorescence	-	+	+	R	R	-	+	-	+	+	-	-	-	*Pseudomonas spp* (possibly *P. aeruginosa*)
7	MPY_0007	DMC2301008	Pus	Yellow, mucoid	Fluorescence	-	+	+	R	R	-	+	-	+	+	-	-	-	*Pseudomonas spp* (possibly *P. aeruginosa*)
8	MPY_0008	DMC2301009	Wound Swab	Green, Mucoid	Fluorescence	-	+	+	R	R	-	+	-	+	+	-	-	-	*Pseudomonas spp* (possibly *P. aeruginosa*)
9	MPY_0009	DMC2301010	Wound Swab	Yellow, mucoid	Fluorescence	-	+	+	R	R	-	+	-	+	+	-	-	-	*Pseudomonas spp* (possibly *P. aeruginosa*)
10	MPY_0010	DMC2301012	Pus	Yellow, mucoid	Fluorescence	-	+	+	R	R	-	+	-	+	+	-	-	-	*Pseudomonas spp* (possibly *P. aeruginosa*)
11	MPY_0011	DMC2301013	Sputum	Yellow, mucoid	Fluorescence	-	+	+	R	R	+	+	-	+	+	-	-	-	*Pseudomonas spp* (possibly *P. aeruginosa*)
12	MPY_0012	DMC2301014	Tracheal Aspirate	Green, Mucoid	Fluorescence	-	+	+	R	R	-	+	-	+	+	-	-	-	*Pseudomonas spp* (possibly *P. aeruginosa*)
13	MPY_0013	DMC2301015	Sputum	Green, Dry, Flat	Fluorescence	-	+	+	Y	R	-	-	-	+	-	-	-	-	*Pseudomonas spp* (possibly *P. putida*)
14	MPY_0014	DMC2301016	Sputum	Yellow, mucoid	Fluorescence	-	+	+	R	R	-	+	-	+	+	-	-	-	*Pseudomonas spp* (possibly *P. aeruginosa*)
15	MPY_0015	DMC2301018	Tracheal Aspirate	Yellow, mucoid	Fluorescence	-	+	+	R	R	+	+	-	+	+	-	-	+	*Pseudomonas spp* (possibly *P. aeruginosa*)
16	MPY_0016	DMC2301019	Wound Swab	Yellow, mucoid	Fluorescence	-	+	+	R	R	-	+	-	+	+	-	-	-	*Pseudomonas spp* (possibly *P. aeruginosa*)
17	MPY_0017	DMC2301020	Tracheal Aspirate	Yellow, mucoid	Fluorescence	-	+	+	R	R	-	+	-	+	+	-	-	-	*Pseudomonas spp* (possibly *P. aeruginosa*)
18	MPY_0018	DMC2301021	Tracheal Aspirate	Yellow, mucoid	Fluorescence	-	+	+	R	R	-	+	-	+	+	-	-	-	*Pseudomonas spp* (possibly *P. aeruginosa*)
19	MPY_0019	DMC2301022	Wound Swab	Green, Dry, Flat	Fluorescence	-	+	+	Y	R	-	-	-	+	-	-	-	-	*Pseudomonas spp* (possibly *P. putida*)
20	MPY_0020	DMC2301023	Wound Swab	Yellow, mucoid	Fluorescence	-	+	+	R	R	-	+	-	+	+	-	-	-	*Pseudomonas spp* (possibly *P. aeruginosa*)

21	MPY_0021	DMC2301024	Sputum	Yellow, mucoid	Fluorescence	-	+	+	R	R	+	+	+	+	+	-	-	-	*Pseudomonas spp* (possibly *P. aeruginosa*)

22	MPY_0022	DMC2301025	Sputum	Green, Dry, Flat	Fluorescence	-	+	+	Y	R	-	-	-	+	-	-	-	-	*Pseudomonas spp* (possibly *P. putida*)
23	MPY_0023	DMC2301026	Wound Swab	Yellow, mucoid	Fluorescence	-	+	+	R	R	+	+	-	+	+	-	-	-	*Pseudomonas spp* (possibly *P. aeruginosa*)
24	MPY_0024	DMC2302001	Urine	Green, Mucoid	Fluorescence	-	+	+	R	R	---	+	-	+	+	-	-	-	*Pseudomonas spp* (possibly *P. aeruginosa*)
25	MPY_0025	DMC2302002	Tracheal Aspirate	Yellow, mucoid	Fluorescence	-	+	+	R	R	-	+	-	+	+	-	-	+	*Pseudomonas spp* (possibly *P. aeruginosa*)
26	MPY_0026	DMC2302003	Pus	Yellow, mucoid	Fluorescence	-	+	+	R	R	-	+	-	+	+	+	-	+	*Pseudomonas spp* (possibly *P. aeruginosa*)
27	MPY_0027	DMC2302004	Wound Swab	Yellow, mucoid	Fluorescence	-	+	+	R	R	-	+	-	+	+	-	-	-	*Pseudomonas spp* (possibly *P. aeruginosa*)
28	MPY_0028	DMC2302005	Wound Swab	Yellow, mucoid	Fluorescence	-	+	+	R	R	-	+	-	+	+	-	-	-	*Pseudomonas spp* (possibly *P. aeruginosa*)
29	MPY_0029	DMC2302006	Wound Swab	Yellow, mucoid	Fluorescence	-	+	+	R	R	-	+	+	+	+	-	-	+	*Pseudomonas spp* (possibly *P. aeruginosa*)
30	MPY_0030	DMC2302007	Urine	Green, Mucoid	Fluorescence	-	+	+	R	R	-	+	-	+	+	-	-	-	*Pseudomonas spp* (possibly *P. aeruginosa*)
31	MPY_0031	DMC2302008	Tracheal Aspirate	Yellow, mucoid	Fluorescence	-	+	+	R	R	-	+	+	+	+	-	-	-	*Pseudomonas spp* (possibly *P. aeruginosa*)
32	MPY_0032	DMC2302009	Tracheal Aspirate	No Pigment	No Fluorescence	-	+	-	Y	Y	+	+	+	-	+	-	+	-	*Klebsiella spp*
33	MPY_0033	DMC2302011	Wound Swab	Yellow, mucoid	Fluorescence	-	+	+	R	R	-	+	-	+	+	+	-	-	*Pseudomonas spp* (possibly *P. aeruginosa*)
34	MPY_0034	DMC2302013	Wound Swab	Green, Mucoid	Fluorescence	-	+	+	R	R	-	+	-	+	-	-	-	-	*Pseudomonas spp* (possibly *P. aeruginosa*)
35	MPY_0035	DMC2302014	Urine	Yellow, mucoid	Fluorescence	-	+	+	R	R	-	+	-	+	+	-	-	-	*Pseudomonas spp* (possibly *P. aeruginosa*)
36	MPY_0036	DMC2302015	Urine	Yellow, mucoid	Fluorescence	-	+	+	R	R	-	+	-	+	+	-	-	-	*Pseudomonas spp* (possibly *P. aeruginosa*)
37	MPY_0037	DMC2302016	Tracheal Aspirate	Green, Dry, Flat	Fluorescence	-	+	+	Y	R	-	-	-	+	-	-	-	-	*Pseudomonas spp* (possibly *P. putida*)
38	MPY_0038	DMC2302018	Tracheal Aspirate	Yellow, mucoid	Fluorescence	-	+	+	R	R	+	+	-	+	+	-	-	-	*Pseudomonas spp* (possibly *P. aeruginosa*)
39	MPY_0039	DMC2302020	Wound Swab	Yellow, mucoid	Fluorescence	-	+	+	R	R	+	+	-	+	+	+	-	-	*Pseudomonas spp* (possibly *P. aeruginosa*)
40	MPY_0040	DMC2302022	Wound Swab	Yellow, mucoid	Fluorescence	-	+	+	R	R	+	+	-	+	+	-	-	-	*Pseudomonas spp* (possibly *P. aeruginosa*)
41	MPY_0041	DMC2302023	Tracheal Aspirate	Yellow, mucoid	Fluorescence	-	+	+	R	R	+	+	-	+	+	-	-	-	*Pseudomonas spp* (possibly *P. aeruginosa*)
42	MPY_0042	DMC2302024	Wound Swab	Green, Mucoid	Fluorescence	-	+	+	R	R	+	+	-	+	+	+	-	-	*Pseudomonas spp* (possibly *P. aeruginosa*)

43	MPY_0043	DMC2303003	Tracheal Aspirate	Yellow, mucoid	Fluorescence	-	+	+	R	R	-	+	-	+	+	+	-	-	*Pseudomonas spp* (possibly *P. aeruginosa*)
44	MPY_0044	DMC2303004	Urine	No Pigment	No Fluorescence	-	+	-	R	Y	-	-	+	-	+	+	-	+	*Proteus spp*
45	MPY_0045	DMC2303005	Wound Swab	Yellow, mucoid	Fluorescence	-	+	+	R	R	-	+	-	+	+	+	-	-	*Pseudomonas spp* (possibly *P. aeruginosa*)
46	MPY_0046	DMC2303006	Sputum	Green, Mucoid	Fluorescence	-	+	+	R	R	-	+	-	+	+	-	-	-	*Pseudomonas spp* (possibly *P. aeruginosa*)
47	MPY_0047	DMC2303007	Wound Swab	Yellow, mucoid	Fluorescence	-	+	+	R	R	-	+	-	+	+	-	-	-	*Pseudomonas spp* (possibly *P. aeruginosa*)
48	MPY_0048	DMC2303008	Wound Swab	Yellow, mucoid	Fluorescence	-	+	+	R	R	-	+	-	+	+	-	-	-	*Pseudomonas spp* (possibly *P. aeruginosa*)
49	MPY_0049	DMC2303009	Wound Swab	Yellow, mucoid	Fluorescence	-	+	+	R	R	-	+	-	+	+	-	-	-	*Pseudomonas spp* (possibly *P. aeruginosa*)
50	MPY_0050	DMC2303010	Pus	Yellow, mucoid	Fluorescence	-	+	+	R	R	-	+	-	+	+	-	-	-	*Pseudomonas spp* (possibly *P. aeruginosa*)
51	MPY_0051	DMC2303011	Wound Swab	Yellow, mucoid	Fluorescence	-	+	+	R	R	-	+	-	+	+	-	-	-	*Pseudomonas spp* (possibly *P. aeruginosa*)
52	MPY_0052	DMC2303012	Tracheal Aspirate	Yellow, mucoid	Fluorescence	-	+	+	R	R	-	+	-	+	+	-	-	-	*Pseudomonas spp* (possibly *P. aeruginosa*)
53	MPY_0053	DMC2303013	Sputum	Green, Mucoid	Fluorescence	-	+	+	R	R	-	+	-	+	+	-	-	-	*Pseudomonas spp* (possibly *P. aeruginosa*)
54	MPY_0054	DMC2303014	Sputum	Yellow, mucoid	Fluorescence	-	+	+	R	R	-	+	-	+	+	-	-	-	*Pseudomonas spp* (possibly *P. aeruginosa*)
55	MPY_0055	DMC2303015	Pus	Yellow, mucoid	Fluorescence	-	+	+	R	R	-	+	-	+	+	-	-	-	*Pseudomonas spp* (possibly *P. aeruginosa*)
56	MPY_0056	DMC2303016	Urine	Green, Mucoid	Fluorescence	-	+	+	R	R	-	+	-	+	+	-	-	-	*Pseudomonas spp* (possibly *P. aeruginosa*)
57	MPY_0057	DMC2303017	Wound Swab	Yellow, mucoid	Fluorescence	-	+	+	R	R	-	+	-	+	+	-	-	-	*Pseudomonas spp* (possibly *P. aeruginosa*)
58	MPY_0058	DMC2304001	Wound Swab	Yellow, mucoid	Fluorescence	-	+	+	R	R	-	+	-	+	+	-	-	-	*Pseudomonas spp* (possibly *P. aeruginosa*)
59	MPY_0059	DMC2304002	Wound Swab	Yellow, mucoid	Fluorescence	-	+	+	R	R	-	+	-	+	+	-	-	-	*Pseudomonas spp* (possibly *P. aeruginosa*)
60	MPY_0060	DMC2304003	Wound Swab	Green, Mucoid	Fluorescence	-	+	+	R	R	-	+	-	+	+	-	-	-	*Pseudomonas spp* (possibly *P. aeruginosa*)
61	MPY_0061	DMC2304004	Pus	Yellow, mucoid	Fluorescence	-	+	+	R	R	-	+	-	+	+	-	-	-	*Pseudomonas spp* (possibly *P. aeruginosa*)
62	MPY_0062	DMC2304005	Sputum	Green, Dry, Flat	Fluorescence	-	+	+	Y	R	-	-	-	+	-	-	-	-	*Pseudomonas spp* (possibly *P. putida*)
63	MPY_0063	DMC2304006	Urine	No Pigment	No Fluorescence	-	+	-	Y	Y	+	+	-	-	+	-	+	-	*Enterobacter app*

64	MPY_0064	DMC2304007	Sputum	Yellow, mucoid	Fluorescence	-	+	+	R	R	-	+	-	+	+	-	-	-	*Pseudomonas spp* (possibly *P. aeruginosa*)
65	MPY_0065	DMC2304008	Pus	Green, Mucoid	Fluorescence	-	+	+	R	R	-	+	-	+	+	-	-	-	*Pseudomonas spp* (possibly *P. aeruginosa*)
66	MPY_0066	DMC2304009	Tracheal Aspirate	Yellow, mucoid	Fluorescence	-	+	+	R	R	-	+	-	+	+	-	-	-	*Pseudomonas spp* (possibly *P. aeruginosa*)
67	MPY_0067	DMC2304010	Sputum	Yellow, mucoid	Fluorescence	-	+	+	R	R	-	+	-	+	+	-	-	-	*Pseudomonas spp* (possibly *P. aeruginosa*)
68	MPY_0068	DMC2304011	Urine	Green, Mucoid	Fluorescence	-	+	+	R	R	-	+	-	+	+	-	-	-	*Pseudomonas spp* (possibly *P. aeruginosa*)
69	MPY_0069	DMC2304012	Wound Swab	Yellow, mucoid	Fluorescence	-	+	+	R	R	-	+	-	+	+	-	-	-	*Pseudomonas spp* (possibly *P. aeruginosa*)
70	MPY_0070	DMC2304013	Pus	Yellow, mucoid	Fluorescence	-	+	+	R	R	-	+	-	+	+	-	-	-	*Pseudomonas spp* (possibly *P. aeruginosa*)
71	MPY_0071	DMC2304014	Wound Swab	Green, Mucoid	Fluorescence	-	+	+	R	R	-	+	-	+	+	-	-	-	*Pseudomonas spp* (possibly *P. aeruginosa*)
72	MPY_0072	DMC2305001	Sputum	Green, Mucoid	Fluorescence	-	+	+	R	R	-	+	-	+	+	-	-	-	*Pseudomonas spp* (possibly *P. aeruginosa*)
73	MPY_0073	DMC2305002	Sputum	Yellow, mucoid	Fluorescence	-	+	+	R	R	-	+	-	+	+	-	-	-	*Pseudomonas spp* (possibly *P. aeruginosa*)
74	MPY_0074	DMC2305003	Wound Swab	Yellow, mucoid	Fluorescence	-	+	+	R	R	-	+	-	+	+	-	-	-	*Pseudomonas spp* (possibly *P. aeruginosa*)
75	MPY_0075	DMC2305004	Wound Swab	Yellow, mucoid	Fluorescence	-	+	+	R	R	-	+	-	+	+	-	-	-	*Pseudomonas spp* (possibly *P. aeruginosa*)
76	MPY_0076	DMC2305005	Tracheal Aspirate	Yellow, mucoid	Fluorescence	-	+	+	R	R	-	+	-	+	+	-	-	-	*Pseudomonas spp* (possibly *P. aeruginosa*)
77	MPY_0077	DMC2305006	Urine	Yellow, mucoid	Fluorescence	-	+	+	R	R	-	+	-	+	+	-	-	-	*Pseudomonas spp* (possibly *P. aeruginosa*)
78	MPY_0078	DMC2305007	Pus	Green, Dry, Flat	Fluorescence	-	+	+	Y	R	-	-	-	+	-	-	-	-	*Pseudomonas spp* (possibly *P. putida*)
79	MPY_0079	DMC2305008	Tracheal Aspirate	Yellow, mucoid	Fluorescence	-	+	+	R	R	-	+	-	+	+	-	-	-	*Pseudomonas spp* (possibly *P. aeruginosa*)
80	MPY_0080	DMC2305009	Sputum	Green, Mucoid	Fluorescence	-	+	+	R	R	-	+	-	+	+	-	-	-	*Pseudomonas spp* (possibly *P. aeruginosa*)
81	MPY_0081	DMC2306002	Sputum	Yellow, mucoid	Fluorescence	-	+	+	R	R	-	+	-	+	+	-	-	-	*Pseudomonas spp* (possibly *P. aeruginosa*)
82	MPY_0082	DMC2306003	Urine	Blue, Mucoid	Fluorescence	-	+	+	R	R	-	+	-	+	+	-	-	-	*Pseudomonas spp* (possibly *P. aeruginosa*)
83	MPY_0083	DMC2306004	Tracheal Aspirate	No Pigment	No Fluorescence	-	+	-	Y	Y	+	+	-	-	+	-	+	-	*Enterobacter spp*
84	MPY_0084	DMC2306005	Urine	No Pigment	No Fluorescence	-	+	-	R	Y	-	-	+	-	+	+	-	+	*Proteus spp*

85	MPY_0085	DMC2306006	Urine	Blue, Mucoid	Fluorescence	-	+	+	R	R	-	+	-	+	+	-	-	-	*Pseudomonas spp* (possibly *P. aeruginosa*)
86	MPY_0086	DMC2306007	Tracheal Aspirate	Blue, Mucoid	Fluorescence	-	+	+	R	R	-	+	-	+	+	-	-	-	*Pseudomonas spp* (possibly *P. aeruginosa*)
87	MPY_0087	DMC2306008	Pus	Green, Mucoid	Fluorescence	-	+	+	R	R	-	+	-	+	+	-	-	-	*Pseudomonas spp* (possibly *P. aeruginosa*)
88	MPY_0088	DMC2306009	Urine	Yellow, mucoid	Fluorescence	-	+	+	R	R	-	+	-	+	+	-	-	-	*Pseudomonas spp* (possibly *P. aeruginosa*)
89	MPY_0089	DMC2307001	Sputum	Green, Mucoid	Fluorescence	-	+	+	R	R	-	+	-	+	+	-	-	-	*Pseudomonas spp* (possibly *P. aeruginosa*)
90	MPY_0090	DMC2307002	Urine	No Pigment	No Fluorescence	-	+	-	Y	Y	+	+	-	-	+	-	+	-	*Enterobacter spp*
91	MPY_0091	DMC2307003	Sputum	Yellow, mucoid	Fluorescence	-	+	+	R	R	-	+	-	+	+	+	-	-	*Pseudomonas spp* (possibly *P. aeruginosa*)
92	MPY_0092	DMC2307006	Urine	Yellow, mucoid	Fluorescence	-	+	+	R	R	-	+	-	+	+	-	-	-	*Pseudomonas spp* (possibly *P. aeruginosa*)
93	MPY_0093	DMC2307007	Sputum	Blue, Mucoid	Fluorescence	-	+	+	R	R	-	+	-	+	+	-	-	-	*Pseudomonas spp* (possibly *P. aeruginosa*)
94	MPY_0094	DMC2307008	Pus	Blue, Mucoid	Fluorescence	-	+	+	R	R	-	+	-	+	+	-	-	-	*Pseudomonas spp* (possibly *P. aeruginosa*)
95	MPY_0095	DMC2307009	Wound Swab	Yellow, mucoid	Fluorescence	-	+	+	R	R	-	+	-	+	+	-	-	-	*Pseudomonas spp* (possibly *P. aeruginosa*)
96	MPY_0096	DMC2307010	Wound Swab	Yellow, mucoid	Fluorescence	-	+	+	R	R	-	+	-	+	+	-	-	-	*Pseudomonas spp* (possibly *P. aeruginosa*)
97	MPY_0097	DMC2307011	Urine	Green, Mucoid	Fluorescence	-	+	+	R	R	-	+	-	+	+	-	-	-	*Pseudomonas spp* (possibly *P. aeruginosa*)
98	MPY_0098	DMC2308001	Tracheal Aspirate	Green, Mucoid	Fluorescence	-	+	+	R	R	-	+	-	+	+	-	-	-	*Pseudomonas spp* (possibly *P. aeruginosa*)
99	MPY_0099	DMC2308002	Sputum	Yellow, mucoid	Fluorescence	-	+	+	R	R	-	+	-	+	+	-	-	-	*Pseudomonas spp* (possibly *P. aeruginosa*)
100	MPY_0100	DMC2308003	Wound Swab	Blue, Mucoid	Fluorescence	-	+	+	R	R	-	+	-	+	+	-	-	-	*Pseudomonas spp* (possibly *P. aeruginosa*)
101	MPY_0101	DMC2308004	Urine	Green, Dry, Flat	Fluorescence	-	+	+	Y	R	-	-	-	+	-	-	-	-	*Pseudomonas spp* (possibly *P. putida*)
102	MPY_0102	DMC2308005	Tracheal Aspirate	Green, Dry, Flat	Fluorescence	-	+	+	Y	R	-	-	-	+	-	-	-	-	*Pseudomonas spp* (possibly *P. putida*)
103	MPY_0103	DMC2308006	Pus	Yellow	Fluorescence	-	+	+	R	R	-	+	-	+	+	-	-	-	*Pseudomonas spp* (possibly *P. aeruginosa*)
104	MPY_0104	DMC2308007	Sputum	Blue, Mucoid	Fluorescence	-	+	+	R	R	-	+	-	+	+	-	-	-	*Pseudomonas spp* (possibly *P. aeruginosa*)
105	MPY_0105	DMC2308008	Wound Swab	Yellow, mucoid	Fluorescence	-	+	+	R	R	-	+	-	+	+	-	-	-	*Pseudomonas spp* (possibly *P. aeruginosa*)

106	MPY_0106	DMC2308009	Urine	Green, Mucoid	Fluorescence	-	+	+	R	R	-	+	-	+	+	-	-	-	*Pseudomonas spp* (possibly *P. aeruginosa*)
107	MPY_0107	DMC2308010	Sputum	No Pigment	No Fluorescence	-	+	-	Y	Y	+	+	-	-	+	-	+	-	*Enterobacter spp*
108	MPY_0108	DMC2308011	Wound Swab	Yellow, mucoid	Fluorescence	-	+	+	R	R	-	+	-	+	+	-	-	-	*Pseudomonas spp* (possibly *P. aeruginosa*)
109	MPY_0109	DMC2308012	Tracheal Aspirate	Yellow, mucoid	Fluorescence	-	+	+	R	R	-	+	-	+	+	-	-	-	*Pseudomonas spp* (possibly *P. aeruginosa*)
110	MPY_0110	DMC2309001	Pus	Yellow, mucoid	Fluorescence	-	+	+	R	R	-	+	-	+	+	-	-	-	*Pseudomonas spp* (possibly *P. aeruginosa*)
111	MPY_0111	DMC2309002	Urine	No Pigment	No Fluorescence	-	+	-	Y	Y	+	+	+	-	+	-	+	-	*Klebsiella spp*
112	MPY_0112	DMC2309003	Tracheal Aspirate	No Pigment	No Fluorescence	-	+	-	Y	Y	+	+	+	-	+	-	+	-	*Klebsiella spp*
113	MPY_0113	DMC2309004	Sputum	Yellow, mucoid	Fluorescence	-	+	+	R	R	-	+	-	+	+	-	-	-	*Pseudomonas spp* (possibly *P. aeruginosa*)
114	MPY_0114	DMC2309006	Tracheal Aspirate	Green, Mucoid	Fluorescence	-	+	+	R	R	-	+	-	+	+	-	-	-	*Pseudomonas spp* (possibly *P. aeruginosa*)
115	MPY_0115	DMC2309007	Wound Swab	Yellow, mucoid	Fluorescence	-	+	+	R	R	-	+	-	+	+	-	-	-	*Pseudomonas spp* (possibly *P. aeruginosa*)
116	MPY_0116	DMC2309010	Sputum	Green, Dry, Flat	Fluorescence	-	+	+	Y	R	-	-	-	+	-	-	-	-	*Pseudomonas spp* (possibly *P. putida*)
117	MPY_0117	DMC2309011	Tracheal Aspirate	Blue, Mucoid	Fluorescence	-	+	+	R	R	-	+	-	+	+	-	-	-	*Pseudomonas spp* (possibly *P. aeruginosa*)
118	MPY_0118	DMC23010001	Wound Swab	Blue, Mucoid	Fluorescence	-	+	+	R	R	-	+	-	+	+	-	-	-	*Pseudomonas spp* (possibly *P. aeruginosa*)
119	MPY_0119	DMC23010002	Wound Swab	Yellow, mucoid	Fluorescence	-	+	+	R	R	-	+	-	+	+	-	-	-	*Pseudomonas spp* (possibly *P. aeruginosa*)
120	MPY_0120	DMC23010003	Wound Swab	Yellow, mucoid	Fluorescence	-	+	+	R	R	-	+	-	+	+	-	-	-	*Pseudomonas spp* (possibly *P. aeruginosa*)
121	MPY_0121	DMC23010004	Tracheal Aspirate	Green, Mucoid	Fluorescence	-	+	+	R	R	-	+	-	+	+	-	-	-	*Pseudomonas spp* (possibly *P. aeruginosa*)
122	MPY_0122	DMC23010005	Urine	Yellow, mucoid	Fluorescence	-	+	+	R	R	-	+	-	+	+	-	-	-	*Pseudomonas spp* (possibly *P. aeruginosa*)
123	MPY_0123	DMC23010006	Pus	No Pigment	No Fluorescence	-	+	-	R	Y	-	-	+	-	+	+	-	+	*Proteus spp*
124	MPY_0124	DMC23010007	Sputum	No Pigment	No Fluorescence	-	+	-	Y	Y	+	+	+	-	+	-	+	-	*Klebsiella spp*
125	MPY_0125	DMC23010008	Tracheal Aspirate	Green, Mucoid	Fluorescence	-	+	+	R	R	-	+	-	+	+	-	-	-	*Pseudomonas spp* (possibly *P. aeruginosa*)
126	MPY_0126	DMC23010009	Urine	Yellow, mucoid	Fluorescence	-	+	+	R	R	-	+	-	+	+	-	-	-	*Pseudomonas spp* (possibly *P. aeruginosa*)

127	MPY_0127	DMC23010010	Tracheal Aspirate	No Pigment	No Fluorescence	-	+	-	Y	Y	+	+	-	-	+	-	+	-	*Enterobacter app*
128	MPY_0128	DMC23010011	Urine	No Pigment	No Fluorescence	-	+	-	Y	Y	+	+	-	-	+	-	+	-	*Enterobacter spp*
129	MPY_0129	DMC23010012	Pus	Yellow, mucoid	Fluorescence	-	+	+	R	R	-	+	-	+	+	-	-	-	*Pseudomonas spp* (possibly *P. aeruginosa*)
130	MPY_0130	DMC23010013	Wound Swab	Blue, Mucoid	Fluorescence	-	+	+	R	R	-	+	-	+	+	-	-	-	*Pseudomonas spp* (possibly *P. aeruginosa*)
131	MPY_0131	DMC23010014	Tracheal Aspirate	Yellow, mucoid	Fluorescence	-	+	+	R	R	-	+	-	+	+	-	-	-	*Pseudomonas spp* (possibly *P. aeruginosa*)
132	MPY_0132	DMC23010017	Pus	Green, Mucoid	Fluorescence	-	+	+	R	R	-	+	-	+	+	-	-	-	*Pseudomonas spp* (possibly *P. aeruginosa*)

KIA= Kligler's Iron Agar
MIU= Motility Indole Urea
NR= Nitrate Reduction
MR= Methyl Red
VP= Voges-Proskauer
S= Slant; B= Butt; G= Gas
R= Red; Y= Yellow
U= Urease; M= Motility
