# Supplementary material for: Multidrug resistance of Pseudomonas aeruginosa: do virulence properties impact on resistance patterns?
Source: Front Microbiol. 2025 Feb 5;16:1508941. doi: 10.3389/fmicb.2025.1508941 (PMC11865748; doi:10.3389/fmicb.2025.1508941)
Supplement: Supplementary file 2 [file Data_Sheet_2.PDF]

Supplementary Table 2: Molecular Identification of Bacterial Isolates based on 16S rRNA Gene Sequencing

Sample ID	Closest blastn match to NCBI Database	E-value	Percent Identity	Molecular Identification
MPY_0002	OR500522.1 *Pseudomonas aeruginosa* strain PC	0.0	99.56%	*P. aeruginosa*
MPY_0007	MK156466.1 *Pseudomonas aeruginosa* PF2	0.0	98.70%	*P. aeruginosa*
MPY_0018	MN490065.1 *Pseudomonas aeruginosa* BUYA-1	0.0	99.87%	*P. aeruginosa*
MPY_0026	OM534570.1 *Pseudomonas aeruginosa* BQ11	0.0	99.93%	*P. aeruginosa*
MPY_0030	OK606067.1 *Pseudomonas aeruginosa* strain ZJI-1	0.0	99.39%	*P. aeruginosa*
MPY_0034	OQ727070.1 *Pseudomonas aeruginosa* WS02	0.0	99.93%	*P. aeruginosa*
MPY_0040	OR500522.1 *Pseudomonas aeruginosa* strain PC	0.0	99.93%	*P. aeruginosa*
MPY_0045	OM818515.1 *Pseudomonas aeruginosa* GBWR9	0.0	99.80%	*P. aeruginosa*
MPY_0052	MW243044.1 *Pseudomonas aeruginosa* 39	0.0	99.93%	*P. aeruginosa*
MPY_0056	OK606067.1 *Pseudomonas aeruginosa* strain ZJI-1	0.0	99.48%	*P. aeruginosa*
MPY_0060	LT797517.1 *Pseudomonas aeruginosa* AT1RP4	0.0	99.93%	*P. aeruginosa*
MPY_0064	KY549641.1 *Pseudomonas aeruginosa* strain Kasamber5	0.0	99.48%	*P. aeruginosa*
MPY_0070	OK217196.1 *Pseudomonas aeruginosa* strain RDF1	0.0	99.48%	*P. aeruginosa*
MPY_0075	KX180920.1 *Pseudomonas aeruginosa* PBS	0.0	99.91%	*P. aeruginosa*
MPY_0082	MT373475.1 *Pseudomonas aeruginosa* NSJ008	0.0	99.93%	*P. aeruginosa*
MPY_0086	NR_026078.1 *Pseudomonas aeruginosa* DSM50071	0.0	99.93%	*P. aeruginosa*
MPY_0091	OQ568312.1 *Pseudomonas aeruginosa* M02	0.0	99.93%	*P. aeruginosa*
MPY_0097	MT300516.1 *Pseudomonas aeruginosa* NPP66	0.0	99.93%	*P. aeruginosa*
MPY_0100	OQ255854.1 *Pseudomonas aeruginosa* AC17	0.0	99.93%	*P. aeruginosa*
MPY_00105	MT771352.1 *Pseudomonas aeruginosa* PF-1	0.0	99.93%	*P. aeruginosa*
MPY_0108	OQ615324.1 *Pseudomonas aeruginosa* SI 1	0.0	99.93%	*P. aeruginosa*
MPY_0115	OQ932907.1 *Pseudomonas aeruginosa* strain FS13	0.0	99.39%	*P. aeruginosa*
MPY_0120	MT000025.1 *Pseudomonas aeruginosa* strain XS 21-2	0.0	99.39%	*P. aeruginosa*
MPY_0126	ON908816.1 *Pseudomonas aeruginosa* strain Pa608	0.0	99.39%	*P. aeruginosa*
MPY_0132	KP282446.1 *Pseudomonas aeruginosa* strain TEN01	0.0	99.39%	*P. aeruginosa*
